# Three novel mutations of *APC* gene in Chinese patients with familial adenomatous polyposis

**DOI:** 10.1007/s13277-016-4986-1

**Published:** 2016-03-22

**Authors:** Qi Liu, Xiaoxia Li, Sen Li, Shengqiang Qu, Yu Wang, Qingzhu Tang, Hongwei Ma, Yang Luo

**Affiliations:** 1 The Research Center for Medical Genomics, MOH Key Laboratory of Cell Biology and Key Laboratory of Medical Cell Biology, Ministry of Education, China Medical University, Shenyang, 110122 China; 2 Department of Gastrointestinal Surgery, Fourth Affiliated Hospital of China Medical University, Shenyang, 110032 China; 3 Department of Developing Pediatrics, Shengjing Hospital, China Medical University, Shenyang, 110004 China

**Keywords:** Familial adenomatous polyposis, *Adenomatous polyposis coli*, Colorectal cancer, Mutation analysis, Immunohistochemistry

## Abstract

Familial adenomatous polyposis (FAP) is an autosomal dominant disorder characterized by the development of hundreds to thousands of colonic adenomas and an increased risk of colorectal cancer. *Adenomatous polyposis coli* (*APC*), encoding a large multidomain protein involved in antagonizing the Wnt signaling pathway, has been identified as the main causative gene responsible for FAP. In this study, we identified three novel mutations as well as two recurrent mutations in the *APC* in five Chinese FAP families by sequencing. Immunohistochemical analysis revealed that among these mutations, a nonsense mutation (c.2510C>G) and two small deletions (c.2016_2047del, c.3180_3184del) led to the truncation of the APC protein and the cytoplasmic and nuclear accumulation of β-catenin in the colorectal samples from affected individuals, respectively. Our study expands the database on mutations of *APC* and provides evidence to understand the function of APC in FAP.

## Introduction

Familial adenomatous polyposis (FAP; OMIM#175100) is an autosomal dominant disorder characterized by hundreds to thousands of adenomatous polyposis throughout the colon and rectum that ultimately evolve into fatal aggressive tumors when left untreated [1]. The penetrance is approximately 100 %, with the appearance of polyps by adolescence or the third decade of life. The incidence of FAP in the population is approximately 1 in 8000. FAP has a relatively equal worldwide distribution and occurs almost equally among males and females. In addition, patients with FAP may have extracolonic manifestation, such as congenital hypertrophy of the retinal pigment epithelium, dental abnormalities, and upper gastrointestinal polyps [2, 3]. In 1991, the *adenomatous polyposis coli* (*APC*) gene was identified as responsible for FAP [4].

*APC* contains 15 exons, is located on chromosome 5q21-q22, and encodes a large multidomain protein composed of 2843 amino acid residues, with a calculated molecular mass of 311.6 kDa [5]. It is ubiquitously expressed in various tissues, especially throughout the large intestine and central nervous system. As a complex functional protein, APC protein combines with glycogen synthesis kinase 3β (GSK-3β) and axin forming a complex called “destruction complex”, which prevents the accumulation of β-catenin by mediating its phosphorylation and degradation in the cytoplasm [6]. Therefore, the APC inactivation leads to the stimulation of the Wnt signaling pathway through decreased degradation of β-catenin. This enables β-catenin to translocate into the nucleus, where it actives transcription factor TCF/LEF to increase the expression of several oncogenes, such as c-myc, cyclin D1, and MMP9 [7]. Hence, *APC* is regarded as a tumor suppressor gene whose inactivation is reported to result in several types of cancer, such as colon and rectum cancer, mammary cancer, and thyroid cancer. Moreover, APC regulates cell migration and adhesion by binding with cytoskeletal proteins including F-actin and microtubules [2]. Furthermore, APC participates in transcriptional activation, apoptosis, DNA repair, and meiosis [2, 8–11].

In this study, we sequenced *APC* in five families with FAP from Chinese Han population and found three novel and two recurrent mutations. Among them, two were nonsense mutations, two were small deletions, and one was a missense mutation. Furthermore, we collected tissue samples from three probands among the affected families and detected the expressions of the truncated APC protein as well as the consequent accumulation of β-catenin to explore potential pathogenic mechanisms.

## Materials and methods

### Sample collection

**Fig. 1:**
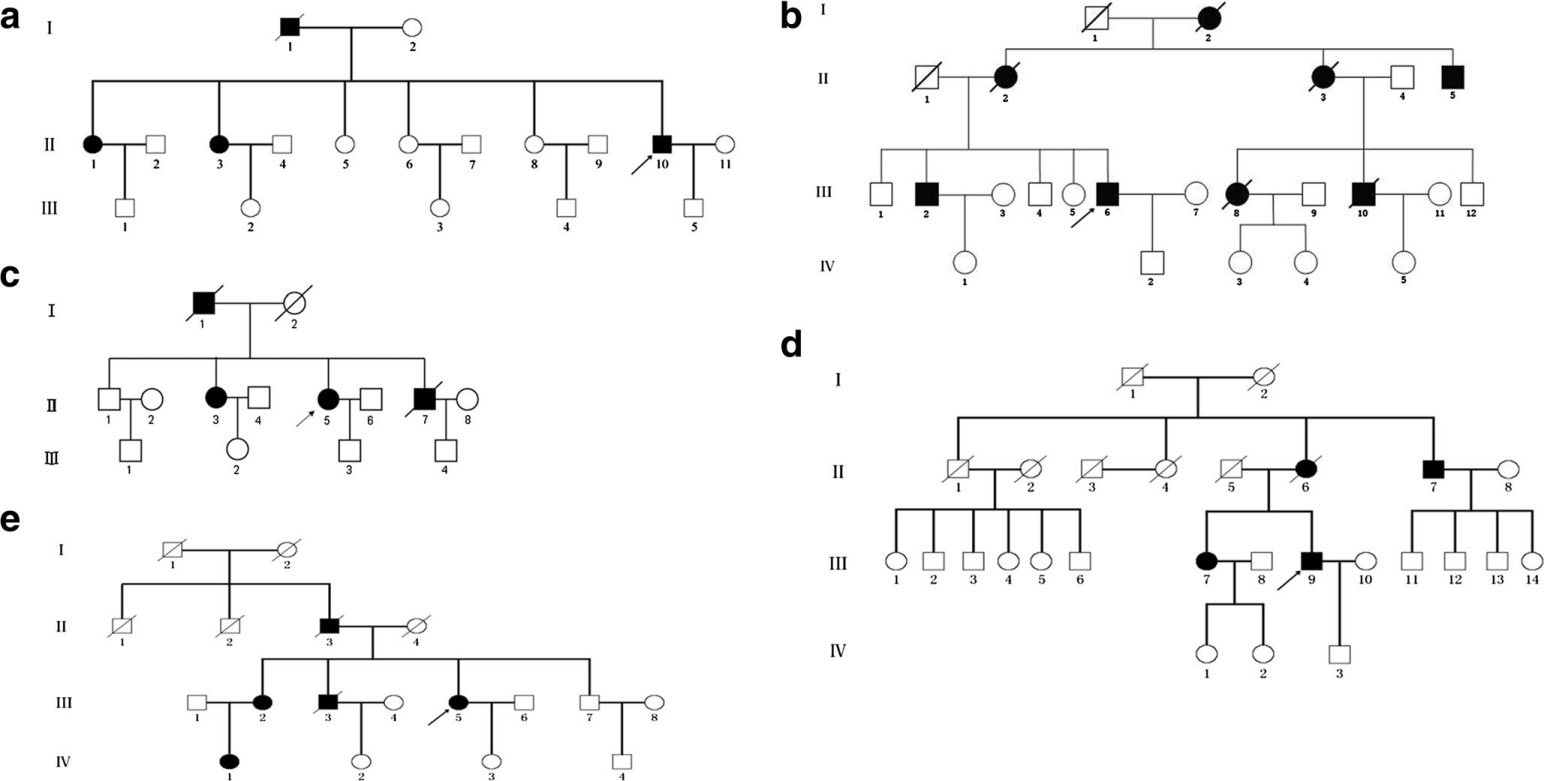
Pedigrees of the five families with FAP. Affected family members are represented by *black symbols*. The probands are indicated with *arrows*

Five probands from five unrelated Chinese families were diagnosed with FAP (Fig. 1). All affected individuals had typical manifestations and family histories as well as presence of numerous polyps under colonoscopy examination. Diagnostic criteria were applied as described previously [12]. The study was approved by the Ethics Committees of China Medical University, and all study subjects gave informed consent.

### Extraction of genomic DNA and PCR

Genomic DNA samples were extracted from peripheral blood samples as described previously [13]. Primers flanking all 15 coding exons and intron-exon boundaries of the *APC* gene were designed. All fragments were amplified as follows: 1 min at 94 °C, 35 cycles (30 s at 94 °C, 30 s at 52–58 °C, 1 min at 72 °C) and 5 min at 72 °C. After amplification, the products were purified on agarose gels and subjected to sequencing [13].

### Immunohistochemistry

Colorectal tissues were obtained from three probands and were paraffin-embedded. Immunohistochemistry was performed in order to determine the expression of APC and β-catenin in the samples. The sections were immunostained using the biotin–avidin–horseradish peroxidase method as described previously and with APC antibody against the C-terminal epitope corresponding to amino acids 2813 to 2823 [14, 15]. Images were visualized and recorded using a Nikon 80i microscope at the indicated magnifications. As controls, normal intestinal tissues were obtained from individuals without FAP.

## Results

### Identification of mutations

**Fig. 2:**
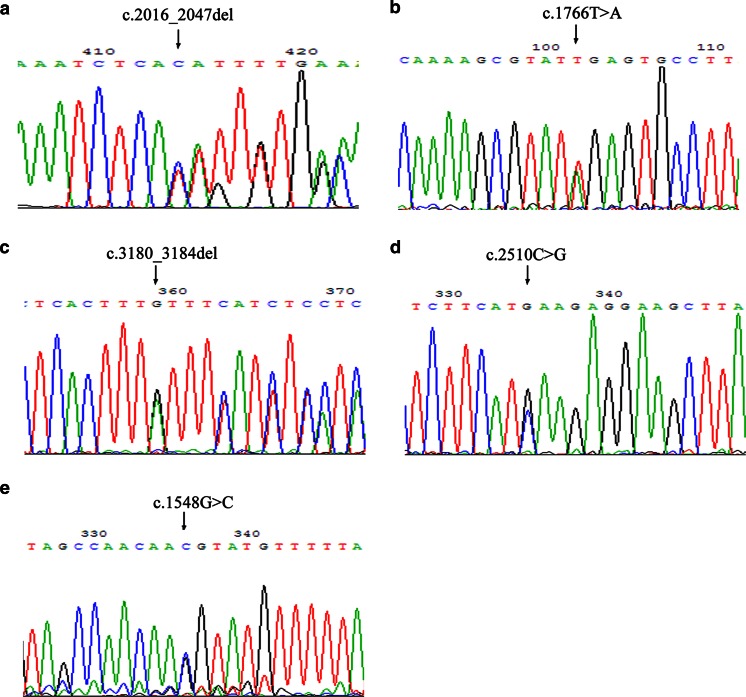
The mutations identified in this study. Direct DNA sequencing showed a nonsense mutation in family 1 (**a**), a missense mutation in family 2 (**b**), a small insertion in family 3 (**c**), a small deletion in family 4 (**d**), and a missense mutation in family 5 (**e**) in the *APC* gene, respectively

Among the five Chinese families with FAP, we found five mutations in *APC* (Table 1). Three of these mutations were novel and two were recurrent. The spectrum of mutations included three substitutions and two small deletions, all of which were heterozygous (Fig. 2a–e).

**Table 1:** Mutations in the *APC* gene detected in this study

Patient	1	2	3	4	5
Sex	Male	Male	Female	Male	Female
Age	34	42	45	55	48
Cancer location	Rectal cancer	Colon cancer	Colorectal cancer	Colorectal cancer	Colorectal cancer
Polyp number	Numerous	Numerous	Numerous	Numerous	Numerous
Nucleotide changes	c.2016_2047del	c.1766T>A	c.3180_3184del	c.2510C>G	c.1548G>C
Amino acid changes	p.Ser673Leufs*10	p.Leu589*	p.Gln1062*	p.Ser837*	p.Lys516Asn
Exon	15	14	15	15	11
Mutation effect	Small deletion	Nonsense	Small deletion	Nonsense	Missense
Novel or recurrent	Novel	Novel	Novel	Recurrent	Recurrent
Truncated protein	Yes	Yes	Yes	Yes	No

In family 1, one 32-bp deletion was detected in exon 15 of *APC* and was defined as c.2016_2047del (Fig. 2a). This mutation was predicted to shift the reading frame from codon 673 and expected to produce a premature termination codon (PTC) at codon 682. In family 2, a nonsense mutation was detected in exon 14 of *APC* and was defined as c.1766 T>A (Fig. 2b). This mutation was predicted to lead to a PTC at codon 589 and to result in truncated APC protein lacking 2254 amino acid residues (Fig. 2b). Moreover, a deletion of the sequence AAAAC was found between c.3180 and c.3184 in family 3, and this 5-bp small deletion was predicted to produce a PTC at codon 1062 (Fig. 2c).

Searching the Human Gene Mutation Database and Colon Cancer Gene Variant Database, we did not find any previous reports about these three variants. Upon restriction endonuclease analysis, all the mutations were segregated in affected individuals from 100 healthy and unrelated normal Chinese subjects, suggesting that neither of them was a polymorphism.

In addition, two recurrent mutations were detected. In family 4, a nonsense mutation, c.2510C>G, was found which was suspected to produce a PTC at codon 837 (Fig. 2d). Moreover, a missense mutation in family 5 replaced a lysine with asparagine at codon 516, which was shown to be a highly conserved residue within the functional domain of the APC (Fig. 2e).

### Molecular analysis of expression patterns

**Fig. 3:**
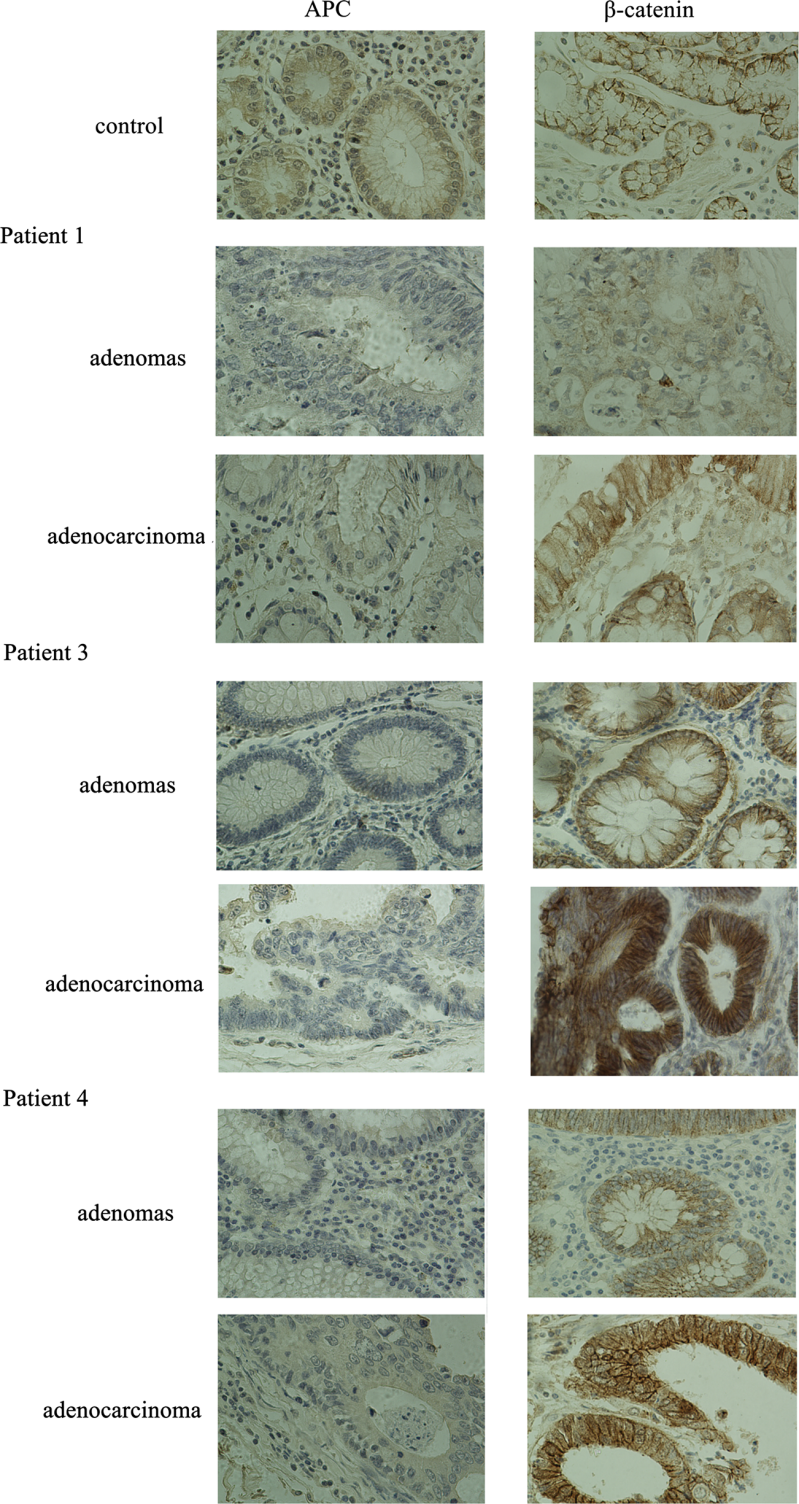
Immunohistochemistry evaluation of the expression of APC and β-catenin in samples from patients 1, 3, and 4 with immunohistochemistry, respectively. Photographs (magnification ×400) of APC (*left*) and β-catenin (*right*) are shown

Immunohistochemistry was performed to analyze the effects of the truncations of APC in paraffin-embedded tissues from families 1, 3, and 4, respectively. As shown in Fig. 3, the expression of APC was positive in control samples but not in the three affected individuals, both in adenoma and adenocarcinoma tissues, supporting the prediction that the truncated APC protein in the affected individuals lacks the C-terminus.

In addition, the expression pattern of β-catenin was also investigated in the samples described above. Higher immunoreactivity of β-catenin was detected in adenocarcinoma samples compared with adenoma in the three paired samples as well as in control tissues. Moreover, β-catenin was mainly localized at the cellular membrane in control tissues while it was mainly localized in the cytoplasm in adenoma samples. Additionally, in adenocarcinoma, a high level of nuclear β-catenin was detected. Notably, the intensity of β-catenin staining was generally consistent with the development from adenomatous polyps to colorectal cancer in each individual.

## Discussion

As a large protein with multiple domains, the human APC contains an oligomerization domain, an armadillo region, three 15-amino acid repeats, seven 20-amino acid repeats, a basic domain, an EB1-binding domain, and a human disc large binding site, which is comprised by amino acids 6-57, 453-767, 1020-1070, 1265-2035, 2200-2400, 2559-2771, and 2771-2843, respectively [2, 16]. The oligomerization domain is responsible for the formation of homodimers, which has been demonstrated to be essential for the activity of the APC protein. It has been suggested that the mutant protein could disturb the activity of wild-type APC by forming heterodimers with it [2]. The armadillo region, as a tightly conserved domain, is linked to protein-protein interactions. Binding partners include APC-stimulated guanine nucleotide exchange factor (Asef1), kinestin-associated protein 3 (SMAP), and APC membrane recruitment protein 1 (AMER1), which suggest that the armadillo domain might be involved in cell-cell adhesion, cell polarization, and migration through cytoskeleton [2]. The armadillo domain is also responsible for the binding of APC with protein phosphatase 2A (PP2A), which represses the Wnt signaling as an antagonist to GSK-3β stabilization [17]. Additionally, APC displays binding sites for β-catenin and axin/conduction at the 15- and 20-amino acid repeats, which are the major domains by which APC regulates the Wnt signaling pathway. In addition, APC can bind to microtubules via the basic domain and EB1-binding domain, which regulate cell migration and cytoskeletal reorganization. Furthermore, the human disc large binding site of APC at the C-terminus might play a negative role in the cell cycle progression from the G_0_/G_1_ to S phase [2].

Most FAP cases are caused by germline mutations in *APC*. Until now, more than 1500 mutations scattered through the 9-kb coding region of the *APC* gene have been reported. Mutations are located primarily at the 5′ end, but extend throughout the length of the *APC* gene. Most of these are point mutations or small deletions/insertions, and most result in N-terminal protein fragments that cannot bind and degrade β-catenin and thus cannot repress Wnt/β-catenin signaling pathway [6–11, 18]. Generally, most mutations are located in exon 15 since it is the largest known exon (6557 bp long). Among the five mutations detected in this study, three mutations were found in this exon. Moreover, loss of heterozygosity and promoter hypermethylation of *APC* has been observed in FAP and colorectal cancer [2, 19–22].

In our study, four mutations, including two small deletions and two nonsense mutations, are expected to result in a truncated APC protein lacking several domains such as β-catenin-binding domains and microtubule-binding domains, which disables the ability of APC to bind to β-catenin and could lead to the underlying the pathogenesis of APC in families 1 to 4. Among these mutations, three were validated as resulting in the loss of the full-length functional APC protein and the accumulation of cytoplasmic and nuclear β-catenin by immunohistochemical staining. However, one mutation could not be validated because samples were not available for analysis. For family 5, the transition of G to C at nucleotide 1548 changes codon 516 from the basic amino acid lysine (AAG) to the neutral amino acid asparagine (AAC) in the highly conserved region of armadillo domain. It is possible that this substitution in a domain involved in protein-protein interactions may change the structural formation of the APC protein, leading to the dominant negative function of the mutant APC, which underlies the pathogenesis of FAP in family 5. Moreover, a large-scale study has clearly addressed the clinical significance of the nuclear translocation of β-catenin with respect to tumor progression, survival, and differential diagnosis [23]. Our results also indicate that nuclear accumulation of β-catenin is correlated with stages of cancer progression in a single individual, which suggests that β-catenin might serve as an additional parameter for disease progression of FAP patients [23].

Furthermore, there appears to be a genotype–phenotype correlation between APC and FAP. For example, mutations between codons 463 to 1444 are generally associated with congenital hypertrophy of the retinal pigment epithelium. Mutations between codons 1445 and 1578 are associated with desmoid tumors, whereas mutations between codons 279 and 1309 correlated with the development of duodenal polyposis [2, 24, 25]. Patients with APC mutations between codons 1249 and 1549 develop polyposis at an early age and exhibit worse survival prognosis while patients with APC mutations in codons 312 to 412 have a later onset of polyposis and exhibit improved survival rates [22]. However, the association between genotype and phenotype and the underlying mechanisms still remain largely unknown. All patients in our study showed no abnormalities other than colorectal adenoma and adenocarcinoma.

Currently, about 35 % of causal mutations in FAP patients could not be identified by Sanger sequencing in *APC* and other polyposis-related genes such as *MUTYH*, *POLD1*, and *POLE*. Similarly, mutations in these patients cannot be detected by fluorescent in situ hybridization (FISH), which is used to detect large chromosomal arrangements in *APC*, suggesting that the known mechanisms leading to FAP are limited [25–28]. Recently, several groups facilitated next-generation sequencing to identify comprehensive causative variants of *APC* such as exon inversion and somatic mosaicism [26–28]. As a powerful high-throughput tool, next-generation sequencing might replace existing screening strategies for polyposis in the future because of its convenience and dramatically decreasing cost [1, 25, 26].

In conclusion, in this study, we identified three novel and two recurrent mutations in the *APC* gene in five Chinese families with FAP and investigated the molecular alterations in tissues from three affected individuals by immunohistochemistry. Together with previous related studies, these results give insight into the still unclearly mechanisms leading to FAP. Additionally, our results can be added to the *APC* mutation spectrum and will contribute further to the understanding of the FAP genotype–phenotype correlations as well as to the pathogenesis of this disease.
